# S15-1 Health-enhancing physical activity: does an active body make an active brain?

**DOI:** 10.1093/eurpub/ckad133.072

**Published:** 2023-09-11

**Authors:** Chair(s):, Greet Cardon, Jannique van Uffelen

**Affiliations:** Ghent University, Belgium; KU Leuven, Belgium

## Abstract

The cognitive health of older adults has become an increasingly important topic as the world's population continues to age. This symposium discusses the latest research and best practices for promoting and maintaining cognitive health in older adults, with an emphasis on the role of physical activity. Four expert presentations will delve into various aspects of this complex topic.

The first presentation will provide a bibliometric analysis of the scientific literature about the link between lifestyle and cognition.

The second presentation will present cross-sectional findings on the relationship between 24-hour movement behaviours and cognition in adults aged 55 years and above.

The third presentation will present the effects and process evaluation of a cognitively enriched walking program for older adults, focusing on the challenges of research in real-life settings.

The final presentation will highlight the impact of a combined physical and cognitive activity program on executive function and walking speed in adults aged 70-85 years.

The bibliometric analysis and cross-sectional findings will provide a broad overview of the current state of research in this area, while the intervention studies will offer practical insights into the design and implementation of physical activity programs that target cognitive health in older adults. This symposium is a valuable opportunity for researchers, practitioners, and policy makers to learn about the latest developments in this field and discuss the future direction of research and policy.

